# Brozopine Inhibits 15-LOX-2 Metabolism Pathway After Transient Focal Cerebral Ischemia in Rats and OGD/R-Induced Hypoxia Injury in PC12 Cells

**DOI:** 10.3389/fphar.2020.00099

**Published:** 2020-02-21

**Authors:** Yuan Gao, Xinyu Cao, Xiaojiao Zhang, Yangjun Wang, He Huang, Yonggang Meng, Junbiao Chang

**Affiliations:** ^1^ Institute of Pharmacology, Zhengzhou University, Henan, China; ^2^ College of Chemistry and Molecular Engineering, Zhengzhou University, Henan, China

**Keywords:** brozopine, middle cerebral artery occlusion, 15-HETE, PC12 cells, OGD/R, inflammation

## Abstract

The goal of this study was to elucidate the mechanisms of protection of Sodium (±)-5-bromo-2-(α-hydroxypentyl) benzoate (trade name: Brozopine, BZP) against cerebral ischemia *in vivo* and *in vitro*. To explore the protective effect of BZP on focal cerebral ischemia-reperfusion injury, we evaluated the effects of various doses of BZP on neurobehavioral score, cerebral infarction volume, cerebral swelling in MCAO rats (ischemia for 2 h, reperfusion for 24 h). In addition, the effects of various doses of BZP on OGD/R-induced-PC12 cells injury (hypoglycemic medium containing 30 mmol Na_2_S_2_O_4_ for 2 h, reoxygenation for 24 h) were evaluated. Four *in vivo* and *in vitro* groups were evaluated to characterize targets of BZP: Control group, Model group, BZP group (10 mg/kg)/BZP group (30 μmol/L), C8E4 group (10 mg/kg)/C8E4 group (30 μmol/L). An ELISA kit was used to determine the levels of 15-HETE (a 15-LOX-2 metabolite) *in vivo* and *in vitro*. Rat nuclear factor κB subunit p65 (NF-κB p65), tumor necrosis factor (TNF-α), interleukin-6 (IL-6), and intercellular adhesion molecule-1 (ICAM-1) were also quantified *in vivo* and *in vitro*. The results showed that BZP improved focal cerebral ischemia-reperfusion injury in rats and PC12 cells treated with Na_2_S_2_O_4_ in dose/concentration-dependent manners through inhibition of production of 15-HETE and expression of NF-κB, IL-6, TNF-α, and ICAM-1. In conclusion, BZP exerted protective effects against cerebral ischemia *via* inhibition of 15-LOX-2 activity.

## Introduction

Cerebral ischemia is a leading cause of death and long-term disability (Hayashi et al., 2015; Benjamin et al., 2018; Pandian et al., 2018), and is the most common neurological disease in the world (Roger et al., 2012). Neuroinflammation and oxidative stress are the main pathological mechanisms of cerebral ischemia-reperfusion, and can lead to neurological dysfunction and brain injury (Zhang et al., 2017). Therefore, anti-inflammatory or antioxidant drugs could be used for treatment of cerebral ischemia-reperfusion injury.

Lipoxygenases (LOX) are non-heme iron enzymes that can oxygenate unsaturated fatty acids such as arachidonic acid (AA) to produce bioactive metabolites. The LOX family of enzymes is characterized by regiospecificity with members including 5-LOX, 8-LOX, 12-LOX and 15-LOX. Two subtypes of 15-LOX have been identified: 15-LOX-1 and 15-LOX-2. The main metabolites of AA produced by the LOX family are 5-, 8-, 12-, and 15-HETE. The levels of free fatty acids (including AA) increase following cerebral ischemia. Increased free AA could result in production of leukotrienes, hydroxy eicosatetraenoic acids, and other bioactive substances produced by 15-LOX. These molecules contribute to pathophysiological processes such as oxidative stress and inflammation after stroke, and can also contribute to apoptosis. Therefore, better understanding of the role 15-LOX after stroke could elucidate novel therapeutic strategies for clinical treatment.

Sodium (±)-5-bromo-2-(α-hydroxypentyl) benzoate (trade name: Brozopine, BZP) is a novel compound with independent intellectual property rights that was synthesized in the College of Chemistry and Molecular Engineering in Zhengzhou University based on 1-3-*n*-Butylphthalide (NBP). BZP has been evaluated in stage II clinical trials and has produced encouraging efficacy results. Our previous studies demonstrated the protective effects of BZP administration by intravenous injection on ischemic stroke in rats. However, the targets of BZP have not been identified.

Initial conceptualization of molecular docking focused on the “lock and key” model. This model states that the first condition for recognition between receptor and ligand is matching of spatial structure. This method was used to establish a three-dimensional structural database of millions of compounds, and then potential ligands were “docked” with the target molecule. Through chemical docking simulation in at the Tsinghua University Academy of Life Sciences, the binding free energies of BZP with 15-LOX-1 and 15-LOX-2 were calculated as −4.1 kcal/mol and −6.2 kcal/mol, indicating strong binding affinity of BZP for 15-LOX-2. 15-LOX-1 was excluded as a possible target protein. Based on these results, we further explored the interactions between BZP and 15-LOX-2, and found that BZP and C8E4, an inhibitor of 15-LOX-2, inhibited the formation of 15-LOX-2 products (HETEs). Therefore, we determined that BZP is an inhibitor of 15-LOX-2, and the mechanism of action of BZP-related protection against cerebral ischemia may be 15-LOX-2–dependent. In this study, we characterized the molecular mechanisms by which BZP protects against cerebral ischemia using *in vivo* and *in vitro* models.

## Materials and Methods

### Animals

SPF male rats (200–250 g) were purchased from Henan Experimental Animal Center, certificate number: SCXK 2017-0001, and housed in a controlled facility with temperature maintained at 22°C–24°C, with free access to food and water.

### Drug Preparation

BZP pellets were synthesized at the College of Chemistry and Molecular Engineering of Zhengzhou University. Each 100-mg pellets contain 10.83 mg BZP. The purity of BZP was 99.4%. BZP was dissolved in DMEM (purchased from Corning Co., Ltd.) to a final concentration of 100 mmol/L, then stored at 4°C until use. C8E4 was purchased from Shanghai Civic Chemical Technology Co., Ltd. It was dissolved DMSO at a concentration of 100 mmol/L and stored at 4°C until use. Stock solutions were diluted with 0.9% saline or DMEM prior to use. Triphenyl chloro tetrazole (2,3,5-triphenyl tetrazolium chloride, TTC) was purchased from Sigma Inc, and dissolved to final concentration of 2% in distilled water prior to use. Nylon thread was purchased from Beijing Shadong Biotechnology Co., Ltd. Paraformaldehyde (4%) was prepared by dissolving 40 g of paraformaldehyde powder in 1000 ml of PBS in a 40°C water bath, then allowed to cool prior to use. Na_2_S_2_O_4_ was purchased from Sigma Co., Ltd. Phosphate buffer (PBS, pH 7.4) was sealed after high pressure sterilization and stored at 4°C. Trypsin was purchased from Solabel Biotechnology Co., Ltd. and stored at 4°C until use.

### Ischemia-Reperfusion Model

A modified Zea-Longa method was used to establish an MCAO model (Longa et al., 1989). Rats were anesthetized with 10% chloral hydrate, then restrained on an isothermal plate to maintain body temperature between 36.5°C and 37.5°C. After disinfection with iodophor, the skin was cut in the middle of the neck and the left common carotid artery was exposed, separation of total neck, external carotid, internal carotid artery, ligation of pterygopalatine artery. At the side of the external carotid artery, a nylon thread plug was inserted from the incision, marked by a forked opening. The thread tie was carefully pushed slowly to a depth of 17 mm, and resistance was felt at about 18 ± 0.5 mm, indicating that the head of the nylon thread had reached the anterior cerebral artery, and the incision was pulled tight and the skin was sutured the skin. After ischemia for 2 h, the rats were anesthetized with ether, and the nylon thread was slowly pulled until the end of the nylon thread ball had retracted to the common carotid artery bifurcation, allowing for reperfusion. Cerebral blood flow dynamics studies were observed by Magnetic Resonance Imaging (MRI).

Before the operation 30 min, BZP was given by oral administration using solid gavage, Sham group and model group were administrated by starch using solid gavage.

### Neurological Behavior Score (Bederson et al., 1986)

Behavioral observation was performed 2 h after ischemia and scored as follows: 0: no symptoms of nerve injury; 1: adductive in the contralateral forelimb; 2: turning to the opposite side while walking; 3: dumping to the opposite side while walking; 4: no spontaneous activity, loss of consciousness. Rats with a score of 0 were considered model failures and were sacrificed.

Inhibition rate = (model group−administration group)/ model group ×100%

### Determination of Cerebral Infarction Volume and Cerebral Swelling by TTC

After 26 h of ischemia, the rats were anesthetized, brains were rapidly removed, and the cerebellum, olfactory bulb, and brain stem, coronal section of 2 mm before and after optic chiasma, each slice was about 2 mm, until the whole rat brain is cut off. The brain was sliced into 2% TTC solution for 10 min. After TTC staining, normal brain tissue was red and infarcted tissue was white. Pictures were taken using a digital camera and infarct area was measured using Photoshop image analysis software. After correction for swelling, the ratio of hemispheric infarct volume (Chen et al., 2012) and swelling ratio (Kollmar et al., 2002) was calculated as follows: infarct volume ratio = [ipsilateral infarction volume-(ipsilateral cerebral volume-contralateral cerebral volume)]/contralateral cerebral volume × 100%

Swelling Ratio = (ipsipelateral brain− contralateral brain volume)/ contralateral brain volume × 100%

### Histopathological Analysis

Briefly, rats (n = 10, for each group)were anesthetized with ether inhalation 26 h after MCAO and then perfused with 0.9% normal saline until the liver turned white, followed by 4% paraformaldehyde solution. The brains were removed and immersed in 4% formaldehyde solution for 24 h at 4°C, dehydrated and embedded in paraffin blocks and then cut into 5-μm-thick coronal sections at room temperature. The sections were deparaffinized and then rehydrated for hematoxylin & eosin (HE) staining. The images were captured by a light microscope(×200), and the analyzer was blind to the studies. Neuronal density was determined by counting the number of necrosis neurons in the cortex, hippocampal and striatum. The results were calculated by dividing the total number of necrosis neurons by the total neuron in 10 fields at random in the cortex, hippocampus and striatum.

### Immunohistochemistry

Following the Behavioral assessment test, the rats' brains were removed and immersed in 4% paraformaldehyde with 1 M phosphate buffer, for 48 h. Therefore it was embedded in paraffin, and five groups of 4- to 6-μm-thick sections were prepared for immunostaining and incubated with primary antibodies against 15-LOX-2, followed by incubation with the appropriate secondary antibody.

The positive cells under the microscope were brown. The percent of immune reaction positive regions in different visual field was measured by image-J analysis. The data is represented by mean ± SD.

### Determination the Percentage of Inhibition of OGD/R Induced PC12 Cell Injury

PC12 cells (1×10^5^/ml) were inoculated in 96-well plate with 100 μL per well. PC12 cells were subjected to hypoxia through incubation with hypoglycemic medium containing 30 mmol of Na_2_S_2_O_4_ for 2 h, then reoxygenated for 24 h, resulting in establishment of a stable OGD/R damage model in PC12 cells.

PC12 experiments were divided into the following 9 groups: Control group, OGD/R group, BZP (5, 10, 15, 20, 30, 40 µmol/L) group and C8E4 (30 µmol/L) group. Following treatment, the cells were cultured in a 5% CO_2_ incubator at 37°C for 24 h, after which the medium was exchanged for serum-free medium. The cells were then incubated in a 5% CO_2_ incubator at 37°C for 2 h following addition of 10 μL of CCK8. Absorbance was determined at 450 nm using an enzyme labeling instrument (Bio-Tek ELX800, USA). The BZP and C8E4 groups were pretreated with BZP and C8E4 for 24 h prior OGD/R damage. Cell damage inhibition rates (%) were calculated using the following formula:

Percentage of inhibition of cell injury= [(A450 of BZP or C8E4‐treated sample−A450 of OGD/R‐treated)/(A450 of OGD−A450 of control/R‐treated)×100%

### Determination of 15-HETE Content

Enzyme-linked immunosorbent assay (ELISA) method was used to determine 15-HETE levels in the brain homogenates and cell supernatants (Yu et al., 2015). Purified rat 15-HETE capture antibody. Then 15-HETE standards or rat samples were added to the plates. Following incubation, HRP-labeled antibodies were added to the wells. After formation of the antibody-antigen-enzyme-labeled antibody complex, the substrate TMB was added after thorough washing. The reaction was stopped after 15 min by addition of acid. The absorbance (OD value) was measured at 450 nm using an enzyme standard instrument. 15-HETE levels were determined using a standard curve.

### Determination of NF-κB P65, TNF- α, IL-6, and ICAM-1 Content

NF-κB p65, TNF-α, IL-6, and ICAM-1 expression were determined in brain homogenates and cell lysates using ELISA (Afshari et al., 2005; Wen et al., 2007; Tien et al., 2016). Rat anti-NF-κB p65, anti-TNF-α, anti-IL-6, and anti-ICAM-1 antibodies were coated on the ELISA plates. Then, rat homogenate samples or cell culture lysates were added to the wells for conjugation to the capture antibodies. After incubation, the wells were washed. Biotinylated rat anti-NF-κB p65, anti-TNF-α, anti-IL-6, and anti-ICAM-1 antibodies and peroxidase labeled avidin were added to the wells. TMB was added to each well. After incubation, the reaction was terminated. Absorbance of the wells was measured at 450 nm and the analytes of interest were quantified using a standard curve.

### Statistical Analysis

One-way analysis of variance was performed using SPSS 17.0. The results are expressed as mean ± standard deviation. If the data were normally distributed and groups had equal variances, we used the LSD test. In the case of unequal variances, we used Dunnett T3 test. *P* < 0.05 indicated statistically significant differences. We used GraphPad Prism 5 software generate graphs.

## Results

### Protective Effect of BZP on Focal Cerebral Ischemia-Reperfusion Injury in Rats

Compared with the sham-operated group, the model group exhibited neurobehavioral deficiencies, as evidenced by contralateral shoulder rotation and forelimb adduction. Contralateral resistance was reduced, walking around, and some animals showed no spontaneous activity, and disturbances in consciousness. The neurobehavioral score of the model group was 3 ± 0.00 (*P* < 0.01) when the shoulders were pushed. Compared with the model group, BZP treatment at 1.2, 2.5, 5, 10, and 20 mg/kg improved ischemia-reperfusion-induced neurobehavioral deficiency, with scores of 2.4 ± 0.52, 1.6 ± 0.52, 1.2 ± 0.42, 1 ± 0.00, and 1 ± 0.00, respectively (*P* < 0.05, *P* < 0.01). The inhibition rate was 20%, 46.7%, 60%, 66.7%, and 66.7%, respectively. There was no significant difference between the BZP-treated and model group following 0.6 mg/kg BZP treatment (2.8 ± 0.42). The ED_50_ was = 8.7 mg/kg.

**Figure 1:**
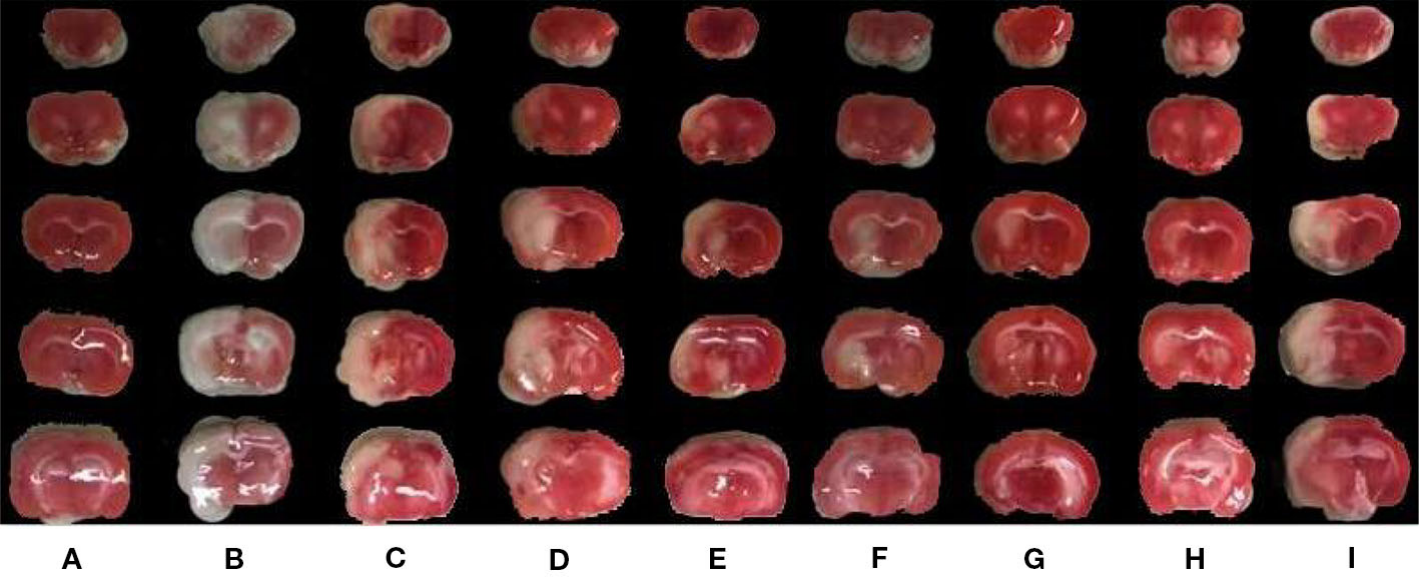
The effects of BZP on infarction volume area of brain in MCAO rats. Note: **(A)** Sham group; **(B)** Model group; **(C)** BZP 0.6 mg/kg group; **(D)** BZP 1.2 mg/kg group; **(E)** BZP 2.5 mg/kg group; **(F)** BZP 5 mg/kg group; **(G)** BZP 10 mg/kg group; **(H)** BZP 20 mg/kg group; **(I)** C8E4 10 mg/kg group. Animals were treated with BZP (0.6, 1.2, 2.5, 5, 10, 20 mg/kg), C8E4 (10 mg/kg) before reperfusion.

As shown in **Figure 1**, BZP treatment at 1.2, 2.5, 5, 10, and 20 mg/kg significantly reduced the cerebral infarct volume compared with the model group (48.5 ± 3.4)%, with reductions to (20.49 ± 2.01)%, (13.88 ± 1.35)%, (9.86 ± 1.68)%, (3.96 ± 0.91)%, and (3.97 ± 1.23)%, respectively (*P* < 0.01). The inhibition rate was 57.8%, 71.4%, 79.7%, 91.8%, and 91.8%, respectively. BZP treatment at 0.6 mg/kg did not significantly protect against ischemia-reperfusion injury (42.8 ± 2.07)% vs. (48.5 ± 3.4)%. The ED_50_ was =1.49 mg/kg. In addition, the model group experienced severe brain swelling (18.57 ± 2.33)%. Compared with the model group, BZP treatment at 1.2, 2.5, 5, 10, and 20 mg/kg significantly reduced brain swelling to (11.86 ± 2.50)%, (6.02 ± 1.95)%, (3.53 ± 0.84)%, (2.22 ± 1.45)%, and (2.14 ± 0.53)%, respectively (*P* < 0.05, *P* < 0.01). The inhibition rate was 36.1%, 67.6%, 81.0%, 88.0%, and 88.5%, respectively. BZP treatment at 0.6 mg/kg (17.44 ± 3.66)% did not differ significantly from the model group. The ED_50_ was =2.93 mg/kg.

**Figure 2:**
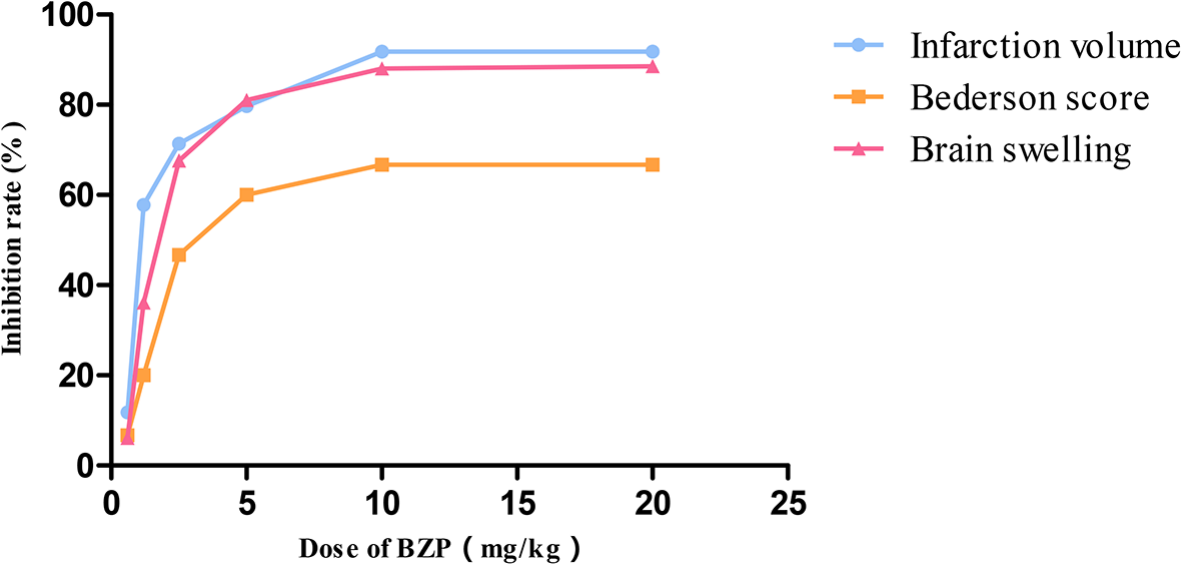
The dose-response curve of Neurologicaldeficit score, infarction volume, and brain swelling after occlusion-reperfusion on post-ischemic treatment with BZP. X bar represents the dose of BZP, Y bar represents the inhibition rate.

BZP pellets treatment at 10 mg/kg) significantly improved nerve loss, reduced the volume of cerebral infarction, and reduced brain edema compared with the C8E4 (10 mg/kg) group [2.8 ± 0.42, (35 ± 8.9)%, (16.65 ± 4.5)%] (*P* < 0.01). Because we were most concerned with infarct volume as an indicator of cerebral ischemia-reperfusion damage, we chose the maximum dose of BZP for subsequent experiments. However, no significant differences were observed between the 20 mg/kg BZP group and the 10 mg/kg BZP group; therefore, 10 mg/kg was used as the maximum effective dose of BZP. (**Table 1**, **Figure 1** and **Figure 2**).

**Table 1:** Effect of BZP on Neurological score, infarction volume, and brain swelling in MCAO rats.

Group	Dose (mg/kg)	Neurological score	Inhibition rate (%)	Infarction volume (%)	Inhibition rate (%)	Swelling (%)	Inhibition rate (%)
Sham		0		0		0	
Model		3 ± 0.00**	0	48.5± 3.4**	0	18.57± 2.33**	0
BZP	0.6	2.8 ± 0.42	6.7	42.8 ± 2.07	11.8	17.44 ± 3.66	6.1
	1.2	2.4 ± 0.52^#^	20	20.49 ± 2.01^##^	57.8	11.86 ± 2.50^#^	36.1
	2.5	1.6 ± 0.52^##^	46.7	13.88 ± 1.35^##^	71.4	6.02 ± 1.95^##^	67.6
	5	1.2 ± 0.42^##^	60	9.86 ± 1.68^##^	79.7	3.53 ± 0.84^##^	81.0
	10	1 ± 0.00^##▲▲^	66.7	3.96 ± 0.91^##▲▲^	91.8	2.22 ± 1.45^##▲▲^	88.0
	20	1 ± 0.00^##^	66.7	3.97 ± 1.23^##^	91.8	2.14 ± 0.53^##^	88.5
C8E4	10	2.8 ± 0.42	6.7	35 ± 8.9	27.8	16.65 ± 4.5	10.3

Animals were treated with BZP (0.6, 1.2, 2.5, 5, 10, 20 mg/kg), C8E4 (10 mg/kg), before reperfusion. Data were presented as mean ± standard deviation. One-way ANOVA test was used to determine statistical significance. **P < 0.01 vs Sham group; ^#^P < 0.05, ^##^P < 0.01 vs Model group; ^▲▲^P < 0.01 vs C8E4 group.

### Histological Alterations

**Figure 3:**
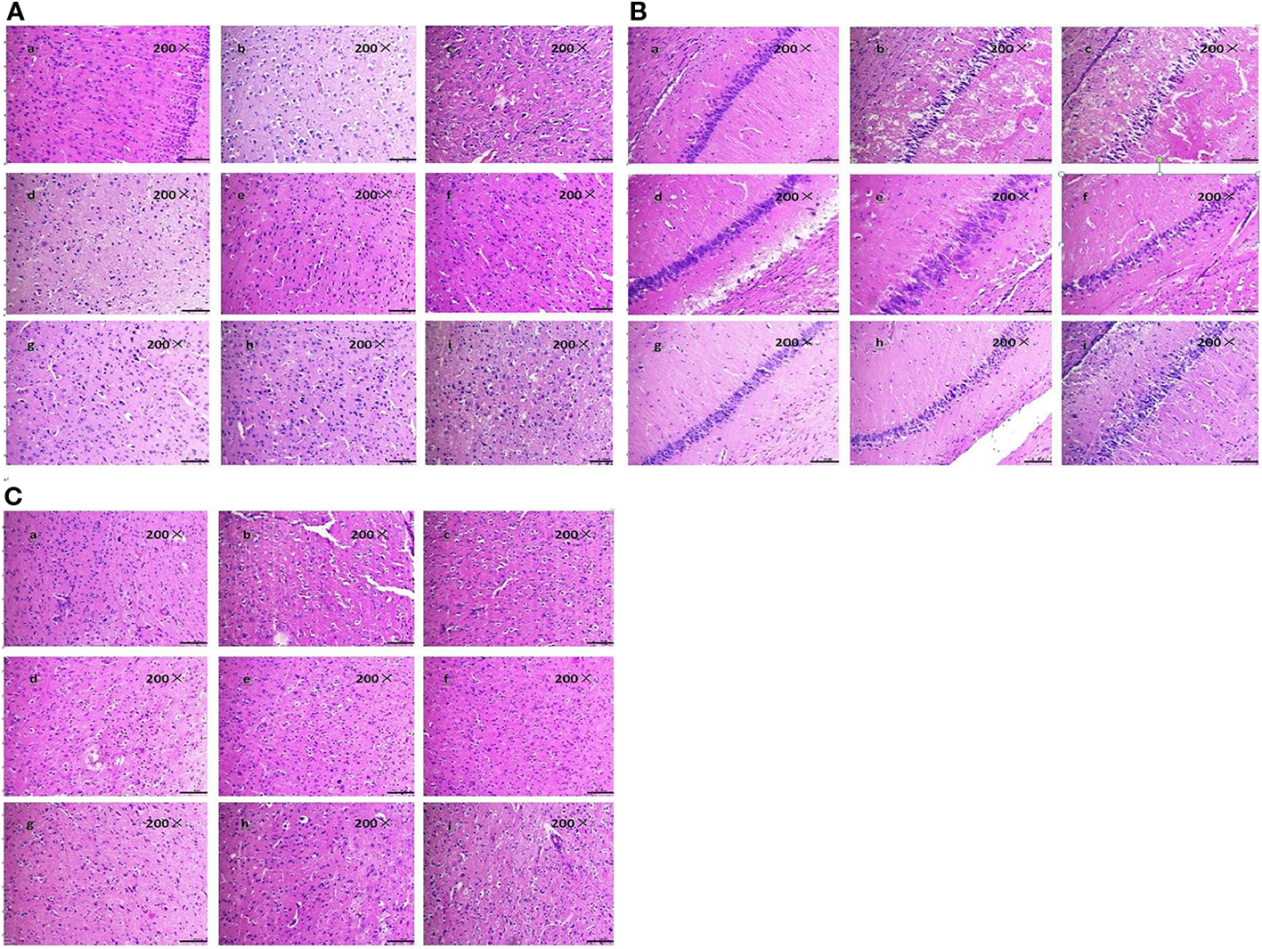
Effect of BZP on Morphological changes in Cortex, Hippocampal and Striatum in MCAO rats under inverted microscope (200×). **(A)** Morphological changes in Cortex; **(B)** Hippocampal; **(C)** Striatum. The groups were as follows: (a) Sham group, (b) Model group, (c) BZP (0.6 mg/kg), (d) BZP (1.2 mg/kg), (e) BZP (2.5 mg/kg), (f) BZP (5 mg/kg), (g) BZP (10 mg/kg), (h) BZP (20 mg/kg), (i) C8E4 (10 mg/kg).

The tissue structure was clear, the cells were dense, arranged neatly, the nucleus was in the middle, the nucleolus was clear, and the cell space was dense and no edema in the sham-operated group in the cortex, hippocampal and striatum. Compared with sham-operated group, marked morphological changes were visualized in ischemic region of model group: neuronal cell loss, nuclei shrinkage, and dark staining of neurons. Compared with model group, BZP (1.2, 2.5, 5, 10, 20 mg/kg) significantly decreased neuronal necrosis [(50.76 ± 3.57) %, (39.31 ± 4.96) %, (29.14 ± 4.96)%, (19.31 ± 4.51)% and (20.97 ± 2.25) % respectively; (*P* < 0.05, *P* < 0.01)] in cortex. Compared with model group, BZP (1.2, 2.5, 5.0, 10, 20 mg/kg) significantly decreased neuronal necrosis [(47.49 ± 9.08) %, (36.29 ± 7.85) %, (24.04 ± 6.42)%, (15.03 ± 2.40)% and (14.20 ± 5.06) % respectively; (*P* < 0.05, *P* < 0.01)] in hippocampal. Compared with model group, BZP (1.2, 2.5, 5.0, 10, 20 mg/kg) significantly decreased neuronal necrosis [(34.57 ± 7.09) %, (27.13 ± 7.21) %, (19.02 ± 5.73)%, (12.32 ± 1.77)% and (12.66 ± 3.87) % respectively; (*P* < 0.05, *P* < 0.01)] in striatum. The results showed BZP had potent protective effect of neurons, the effect of BZP enteric-coated pellets (10 mg/kg) group was better than that of C8E4 group with equal molar dose in the cortex, hippocampal and striatum (*P* < 0.01). (The results were shown in **Table 2**, **Figure 3**.)

**Table 2:** Effect of BZP on density and distribution of necrosis in MCAO rats.

Group	Dose (mg/kg)	n	Cortex (%)	Hippocampal (%)	Striatum (%)
Sham		10	6.33 ± 1.93	5.95 ± 1.64	5.12 ± 1.55
Model		10	60.98 ± 9.29**	57.22 ± 9.10**	41.11 ± 6.43**
BZP	0.6	10	58.23 ± 4.78	55.13 ± 8.45	40.17 ± 6.63
	1.2	10	50.76 ± 3.57^#^	47.49 ± 9.08^#^	34.57 ± 7.09^#^
	2.5	10	39.31 ± 4.96^##^	36.29 ± 7.85^##^	27.13 ± 7.21^##^
	5	10	29.14 ± 2.96^##^	24.04 ± 6.42^##^	19.02 ± 5.73^##^
	10	10	19.31 ± 4.51^##▲▲^	15.03 ±2.40^##▲▲^	12.32 ±1.77^##▲▲^
	20	10	20.97 ± 2.25^##^	14.20 ± 5.06^##^	12.66 ± 3.87^##^
C8E4	10	10	47.98 ± 9.44^##^	44.16 ± 10.7^##^	29.02 ± 4.36^##^

Data were represented by mean ± standard deviation. **P < 0.01 vs Sham group; ^#^P < 0.05, ^##^P < 0.01 vs Model group; ^▲▲^P < 0.01 vs C8E4 group.

### BZP Decreased the Expression of 15-LOX-2 in MCAO Rats

**Figure 4:**
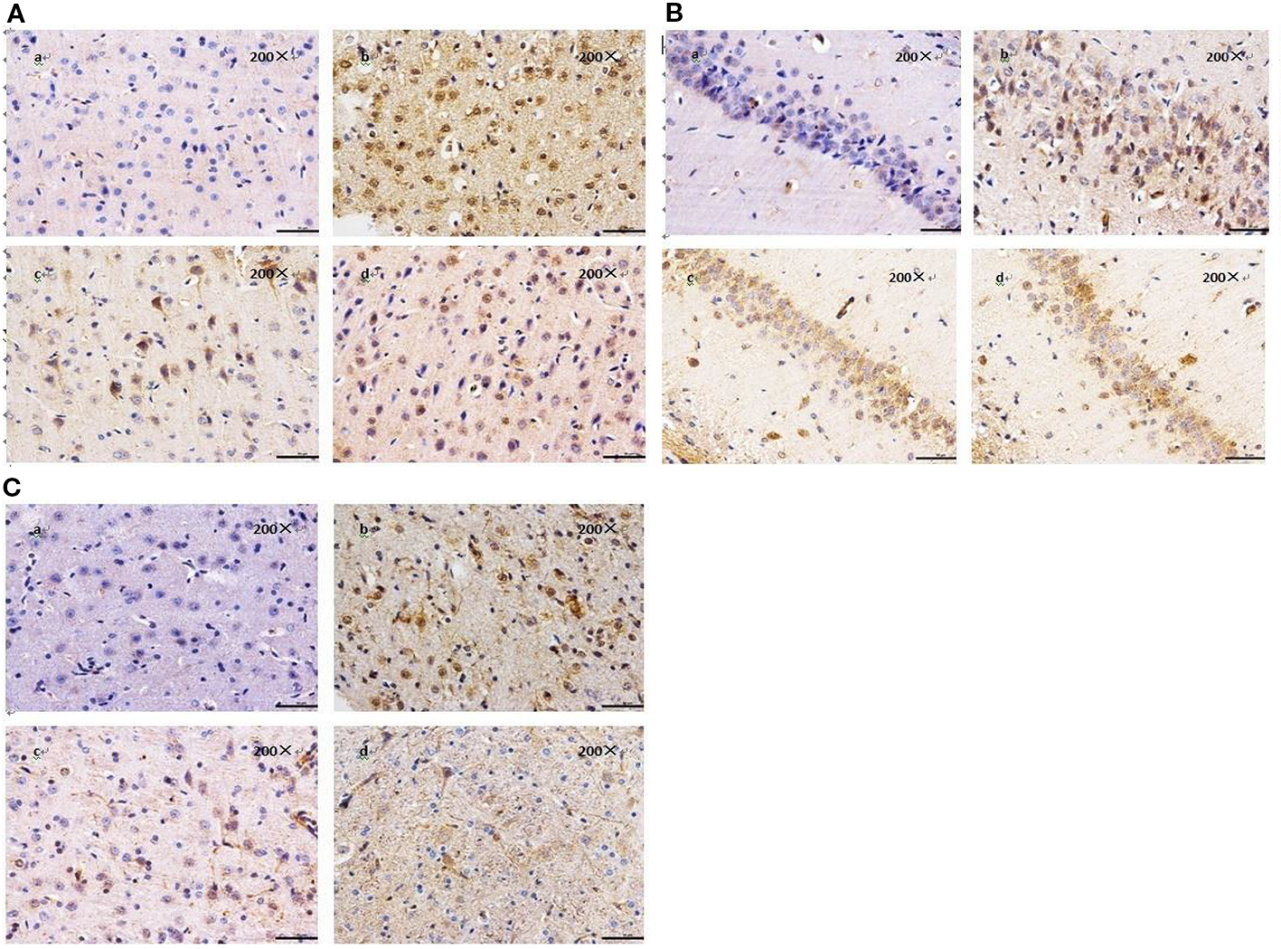
Effect of BZP on the expression of 15-LOX-2 in Cortex, Hippocampal and Striatu in MCAO rats under inverted microscope (200×). **(A)** Morphological changes in Cortex; **(B)** Hippocampal; **(C)** Striatum. The groups were as follows: (a) Sham group, (b) Model group, (c) BZP (10 mg/kg), (d) C8E4 (10 mg/kg).

The results of immunohistochemistry showed that the expression of 15-LOX-2 in cortex, hippocampus and striatum of model group was significantly higher than that of sham group (*P* < 0.01), the expression of 15-LOX-2 in BZP (10 mg/kg) enteric-coated pellets group was significantly lower than that in model group (*P* < 0.01). There was no significant difference in the expression of 15-LOX-2 between 10 mg/kg enteric-coated pellets group and equal molar dose C8E4 group (*P* > 0.05). It was shown that BZP was an inhibitor of 15-LOX-2. (The results were shown in **Table 3**, **Figure 4**.)

**Table 3:** Effect of BZP on the expression of 15-LOX-2 in Cortex, Hippocampal and Striatum in MCAO rats.

Group	Dose (mg/kg)	n	Cortex (%)	Hippocampal (%)	Striatum (%)
Sham		10	15.10 ± 2.95	10.51 ± 2.43	6.55 ± 1.51
Model		10	63.59 ± 3.77**	47.09 ± 5.15**	25.94 ± 6.74**
BZP	10	10	21.62 ± 3.48^##^	17.65 ± 3.81^##^	12.43 ± 2.66^##^
C8E4	10	10	19.77 ± 4.36 ^##^	16.99 ± 4.19 ^##^	10.31 ± 3.12 ^##^

Data were represented by mean ± standard deviation. **P < 0.01 vs Sham group; ^##^P < 0.01 vs Model group.

### BZP Reduce the Content of 15-HETE in MCAO Rats

**Figure 5:**
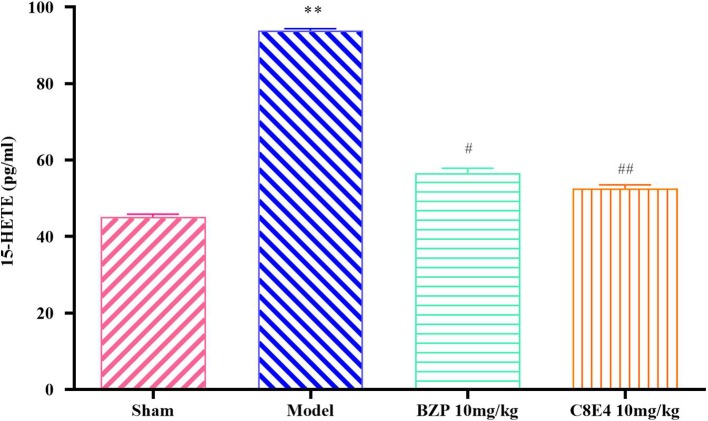
Effect of BZP on 15-HETE levels in MCAO rats. Animals were treated with BZP (10 mg/kg), C8E4 (10 mg/kg) before reperfusion. Data were presented as mean ± sd. One-way ANOVA test was used to determine statistical significance. **P< 0.01 vs Sham group; ^#^ *P*< 0.05, ^##^ *P*< 0.01 vs Model group.

**Figure 5** showed that the content of 15-HETE in the model group was 93.94 ± 0.49 pg/ml, which was significantly higher than that in the sham operation group. Compared with the model group, BZP (10 mg/kg) treatment significantly reduced 15-HETE levels (56.67 ± 1.2 pg/ml; *P* < 0.05). 15-HETE levels in the C8E4 (10 mg/kg) group were significantly decreased (52.65 ± 0.95 pg/ml; *P* < 0.01). No differences were observed in 15-HETE levels between rats administered BZP or C8E4 of equal molar dose (*P* > 0.05).

### BZP Decreased the Expression of NF-κb p65, TNF-α, IL-6, ICAM-1 in MCAO Rats

The expression of NF-κB in the model group (549.79 ± 7.37 pg/ml) was significantly higher than that in the sham operation group. Compared with the model group, the BZP (10 mg/kg) group and the C8E4 (10 mg/kg) group showed decreased expression of NF-κB (104.79 ± 8.59 pg/ml, 206.21 ± 3.13 pg/ml; *P* < 0.01), and BZP treatment resulted in significantly lower expression of NF-κB than treatment with C8E4 (*P* < 0. 05).

The expression of TNF-α in the model group (261.95 ± 1.35 pg/ml) was significantly higher than that in the sham operation group. Compared with the model group, the expression of TNF-α was decreased in the BZP (10 mg/kg) group (109.95 ± 6.36 pg/ml; *P* < 0.05). The expression of TNF-α in the C8E4 (10 mg/kg) group was 197 ± 10.54 pg/ml, which was significantly higher than that in the BZP group (*P* < 0. 05).

The expression of IL-6 in the model group (309.67 ± 6.84 pg/ml) was higher than that in the sham-operation group. Compared with the model group, the expression of IL-6 in the BZP (10 mg/kg) group was significantly lower (161 ± 8.72 pg/ml; *P* < 0.01). The expression of IL-6 in the C8E4 (10 mg/kg) group was 251.83 ± 9.90 pg/ml, which was significantly higher than that in the BZP group (*P* < 0.05).

The expression of ICAM-1 in the model group (1325.21 ± 11.62 pg/ml) was significantly higher than that in the sham-operation group. Compared with the model group, the expression of ICAM-1 in the BZP group was significantly lower (877.14 ± 4.65 pg/ml; *P* < 0.01). The expression of ICAM-1 was also decreased in the C8E4 (10 mg/kg) group (1050.43 ± 0.20 pg/ml; *P* < 0.05), but was significantly higher than that in the BZP group (*P* < 0.05). The results of these experiments were summarized in **Table 4**.

**Table 4:** Effect of BZP on the expression of NF-κB, TNF-α, IL-6, and ICAM-1 in MCAO rats.

Group	Dose (mg/kg)	NF-κB（pg/ml）	TNF-α（pg/ml）	IL-6（pg/ml）	ICAM-1（pg/ml）
Sham		89.5 ± 4.34	94.72 ± 1.29	138.08 ± 1.53	663.36 ± 4.14
Model		549.79 ± 7.37**	261.95 ± 1.35**	309.67 ± 6.84^*^	1325.21 ± 11.62**
BZP	10	104.79 ± 8.59^##▲^	109.95 ± 6.36^#▲^	161 ± 8.72^##▲^	877.14 ± 4.65^##▲^
C8E4	10	206.21 ± 3.13^##^	197 ± 10.54	251.83 ± 9.90	1050.43 ± 0.20^#^

Animals were treated with BZP (10 mg/kg), C8E4 (10 mg/kg) before reperfusion. Data were presented as mean ± standard deviation. One-way ANOVA test was used to determine statistical significance.*P< 0.05, **P< 0.01 vs Sham group; ^#^P< 0.05, ^##^P< 0.01 vs Model group; ^▲^P< 0.05 vs C8E4 group.

### Protective Effect of BZP on PC12 Cell Injury Induced by Na_2_S_2_O_4_

**Figure 6:**
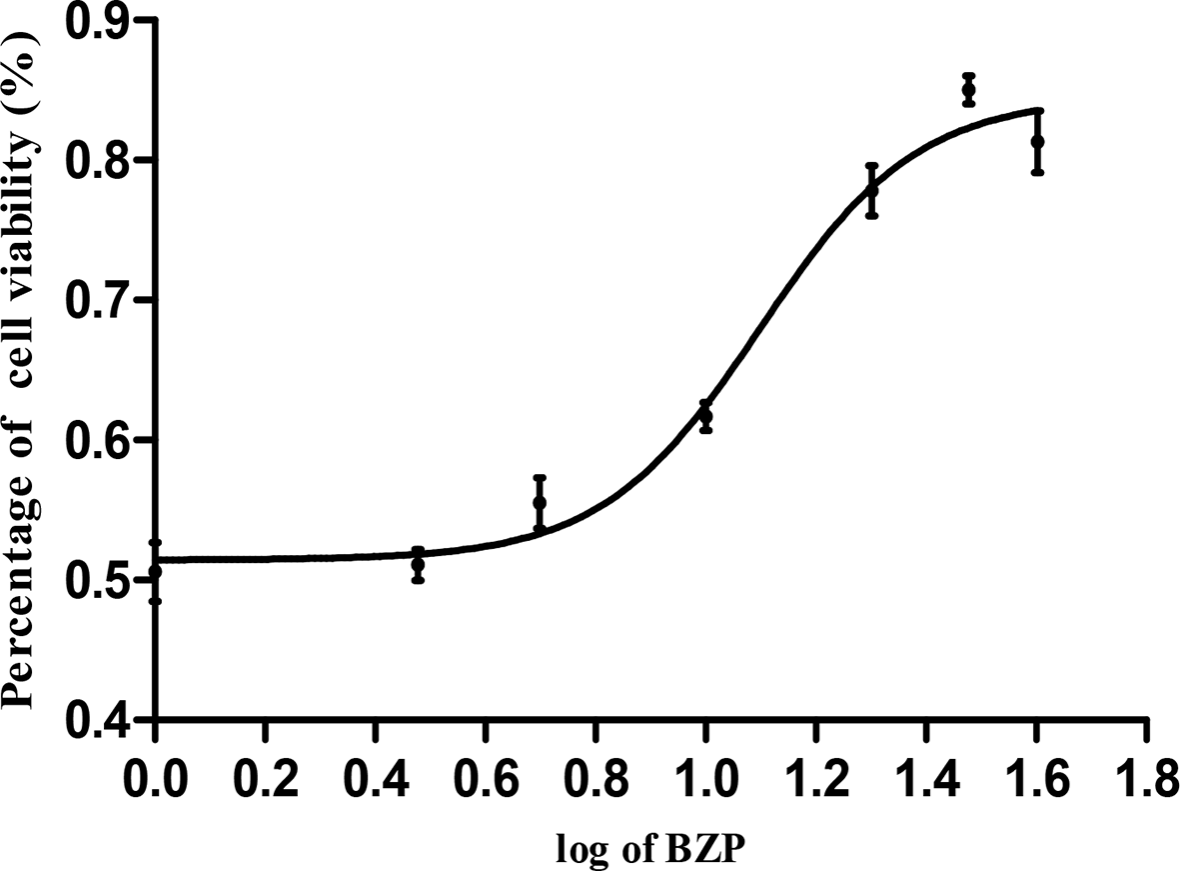
The concentration-response curve of cell viability of PC12 cells induced by OGD/R. PC12 cells were exposed to 30 mmol/L Na_2_S_2_O_4_ with low-glucose DMEM for 2 h and then reoxygenation for 24 h, and also pretreated with BZP (5–40 μmol/L) for 24 h respectively. X bar represents the concentration of BZP, Y bar represents the inhibition rate.

The cell survival rates for each group were summarized in **Table 5** and **Figure 6**. Compared with the Control group, the survival rate of PC12 cells was significantly decreased in the OGD/R injury group (50.6%; *P* < 0.01). The survival rates of PC12 cells pretreated with 5, 10, 15, 20, 30, and 40 µmol/L BZP were significantly different from those in OGD/R injury group (55.5%, 61.7%, 69.6%, 77.8%, and 85%; *P* < 0.05, *P* < 0.01). The EC_50_ was =19 μmol/L. However, the survival rate of PC12 cells pretreated with 40 μmol/L BZP (81.3%) was slightly lower than that of cells treated with 30 μmol/L BZP, suggesting that 30 μmol/L was the maximum effective concentration of BZP. BZP (30 μmol/L) treatment provided better protection than C8E4 (30 μmol/L) treatment (62.8%; *P* < 0.01).

**Table 5:** The Cell Viability of OGD/R induced PC12 cells injury.

Group	Concentration (μmol/L)	A450	Percentage of inhibition of cell injury (%)
Control		0.92 ± 0.01	
OGD/R		0.47 ± 0.02**	50.6
BZP	5	0.51 ± 0.01^#^	55.5
	10	0.57 ± 0.02^##^	61.7
	15	0.64 ± 0.01^##^	69.6
	20	0.72 ± 0.02^##^	77.8
	30	0.78 ± 0.01^##▲▲^	85
	40	0.75 ± 0.02^##^	81.3
C8E4	30	0.58 ± 0.04^##^	62.8

Effects of BZP on cell viability of PC12 cells induced by OGD/R.PC12 cells were exposed to 30 mmol/L Na2S2O4 with low-glucose DMEM for 2 h and then reoxygenation for 24 h, and also pretreated with BZP (5–40 μmol/L) and C8E4 (30 μmol/L) for 24 h respectively. Data were presented as mean ± standard deviation. A one-way analysis of variance (ANOVA) was used to determine if the differences between the groups were statistically significant. **P < 0.01 vs Control group; ^#^P < 0.05, ^##^P < 0.01 vs Model group; ^▲▲^P < 0.01 vs C8E4 group.

### BZP Improved the Morphology of PC12 Cells Treated With Na_2_S_2_O_4_

**Figure 7:**
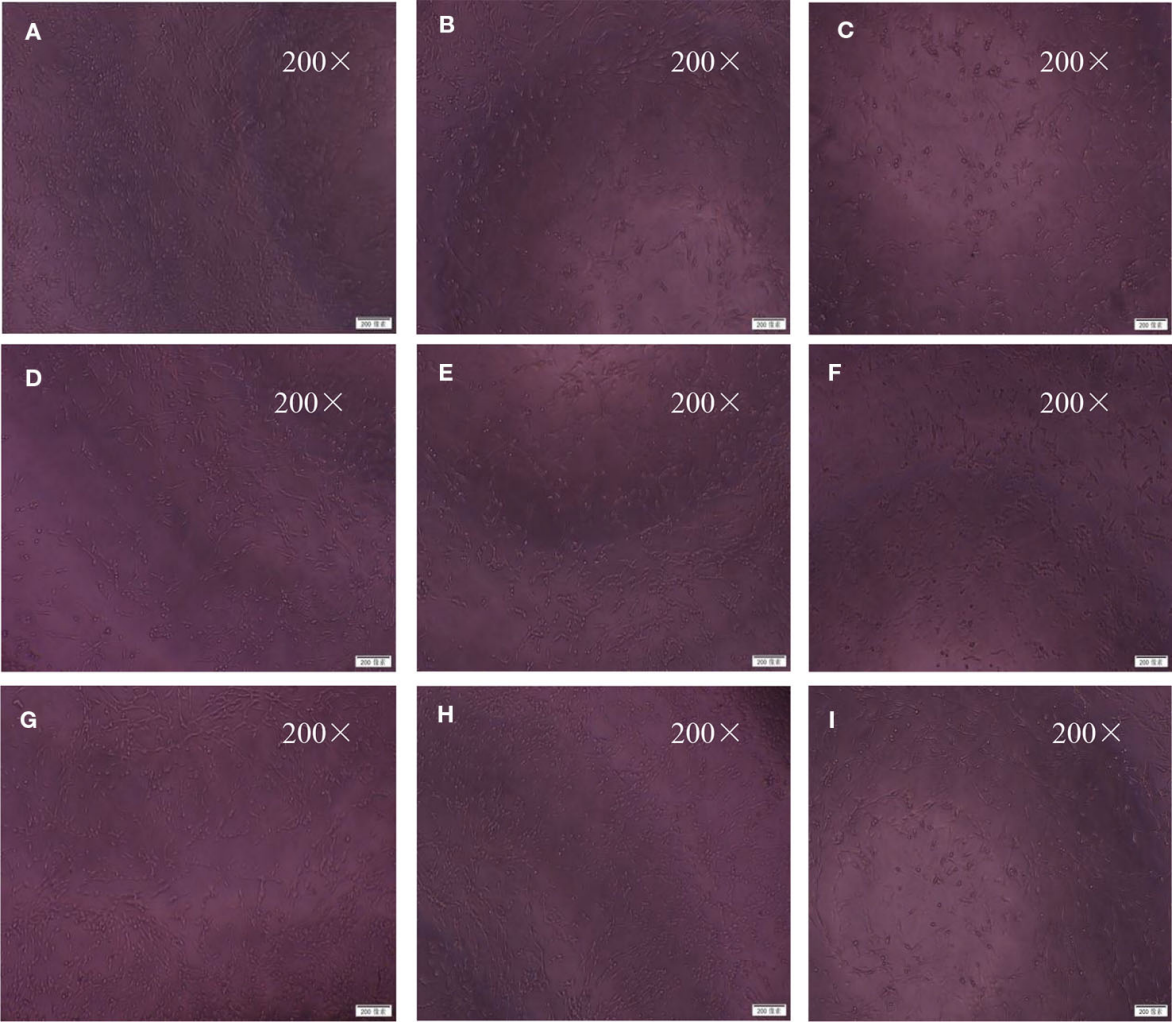
Morphological changes under inverted microscope (200×). The cells were exposed to 30 mmol/L Na_2_S_2_O_4_ with low-glucose DMEM for 2 h after treatment with BZP (5–40 μmol/L) and C8E4 (30 μmol/L) for 24 h, respectively, and then reoxygenation for 24 h. The groups were as follows: **(A)** Control group, **(B)** OGD/R group, **(C)** BZP (5 µmol/L) group, **(D)** BZP (10 µmol/L) group, **(E)** BZP (15 µmol/L) group, **(F)** BZP (20 µmol/L) group, **(G)** BZP (30 µmol/L) group, **(H)** BZP (40 µmol/L) group, **(I)** C8E4 (30 µmol/L) group.

In the Control group, the neuronal bodies were larger, and they had long dendritic protuberances with polygonal or fusiform shapes. while in the OGD/R injured group, the number of nerve cells decreased, the cell bodies shrank, some of the cells did not have processes, and some of the nerve cells were round. After preconditioning of BZP 30 µmol/L, C8E4 30 µmol/L, the injury of nerve cells was improved, the cell body was larger and dendritic process was found. The effect of BZP (30 µmol/L) group was better than C8E4 (30 µmol/L) group. C8E4 (30 µmol/L) and BZP (30 µmol/L) were equal molar dose. (The results were shown in **Figure 7**).

### BZP Reduced 15-HETE Levels in PC12 Cells Treated With Na_2_S_2_O_4_

**Figure 8:**
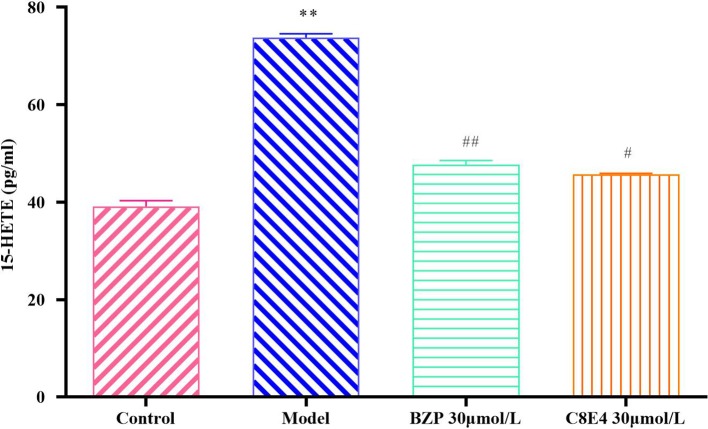
Effect of BZP on 15-HETE levels in PC12 cells induced by OGD/R. PC12 cells were exposed to 30 mmol/L Na_2_S_2_O_4_ with low-glucose DMEM for 2 h and then reoxygenation for 24 h, and also pretreated with BZP (30 μmol/L) and C8E4 (30 μmol/L) for 24 h respectively. Data were presented as mean ± sd. One-way ANOVA test was used to determine statistical significance. **P< 0.01 vs Sham group; ^#^ *P*< 0.05, ^##^ *P*< 0.01 vs Model group.

15-HETE levels in the OGD/R injury group (73.79 ± 0.82 pg/ml) were significantly higher than those in the Control group, and BZP (30 μmol/L) treatment significantly decreased 15-HETE levels (47.71 ± 0.87 pg/ml; *P* < 0.01) compared with the OGD/R group. No significant differences were observed between the BZP (30 μmol/L) group and the C8E4 (30 μmol/L) group (45.79 ± 0.16 pg/ml; *P* > 0.05). (The results were shown in **Figure 8**).

### BZP Decreased the Expression of NF-κb P65, TNF-α, IL-6, and ICAM-1 in PC12 Cells Treated With Na_2_S_2_O_4_

The expression of NF-κB in OGD/R injury group (178.36 ± 1.72 pg/ml) was significantly higher than that in the Control group. BZP (30 μmol/L) and C8E4 (30 μmol/L) treatments both significantly decreased NF-κB expression (104.79 ± 8.59 pg/ml; *P* < 0.01) compared with the OGD/R injury group, and the expression of NF-κB was lower in the C8E4 (30 μmol/L) group (113.43 ± 3.43 pg/ml; *P* < 0.05), but there was no difference in NF-κB expression between the BZP and C8E4 groups (*P* > 0.05).

The expression of TNF-α in the OGD/R injury group (94.82 ± 1.03 pg/ml) was higher than that in the Control group. Compared with the OGD/R injury group, the BZP (30 μmol/L) group showed significantly lower expression of TNF-α (33.68 ± 1.99 pg/ml; *P* < 0.01). Compared with the C8E4 (30 μmol/L) group (76.18 ± 1.67 pg/ml), the BZP (30 μmol/L) group had significantly lower expression of TNF-α (*P* < 0.01).

The expression of IL-6 in the OGD/R injury group (112.92 ± 3.89 pg/ml) was higher than that in the Control group. Compared with the OGD/R group, the BZP (30 μmol/L) group had lower IL-6 content (60.92 ± 1.30 pg/ml; *P* < 0.05). Furthermore, BZP (30 μmol/L) treatment decreased IL-6 expression to a greater extent than C8E4 (30 μmol/L) treatment (98.83 ± 2.60 pg/ml; *P* < 0.05).

The expression of ICAM-1 in the OGD/R injury group (372.5 ± 5.15 pg/ml) was significantly higher than that in the Control group. Both BZP (30 μmol/L) and C8E4 (30 μmol/L) treatments significantly decreased ICAM-1 expression (83.64 ± 4.55 pg/ml, 213.29 ± 2.42 pg/ml); compared with the OGD/R injury group, but BZP reduced ICAM-1 to a significantly greater extent than C8E4 (*P* < 0.01). (The results were shown in **Table 6**).

**Table 6:** Effect of BZP on the expression of NF-κB, TNF-α, IL-6 and ICAM-1 in OGD/R induced PC12 cells injury.

Group	Concentration (μmol/L)	NF-κB （pg/ml）	TNF-α（pg/ml）	IL-6（pg/ml）	ICAM-1（pg/ml）
Control		84.79 ± 0.91	31.77 ± 2.76	33.08 ± 1.06	45.07 ± 9.39
OGD/R		178.36 ± 1.72**	94.82 ± 1.03^*^	112.92 ± 3.89^*^	372.5 ± 5.15**
BZP	30	96 ± 0.61^##^	33.68 ± 1.99^##▲▲^	60.92 ± 1.30^#▲^	83.64 ± 4.55^##▲▲^
C8E4	30	113.43 ± 3.43^#^	76.18 ± 1.67^#^	98.83 ± 2.60	213.29 ± 2.42^##^

Data were presented as mean ± standard deviation. One-way ANOVA test was used to determine statistical significance. *P < 0.05, **P < 0.01 vs Control group; ^#^P < 0.05, ^##^P < 0.01 vs Model group; ^▲^P < 0.05, ^▲▲^P < 0.01 vs C8E4 group.

## Discussion

Cerebral arterial thrombosis is a cerebrovascular disease caused by interruption of blood circulation (Pendlebury and Rothwell, 2009). Although considerable progress had been made in understanding ischemic stroke, development of safer, more effective drugs is of great importance (Khatri et al., 2012; Thompson and Ronaldson, 2014). In our previous study, BZP exerted protective effects against global cerebral ischemic injury in rats. The mechanism of these effects may have been related to inhibition of apoptosis and activation of the survival signaling pathway (Gao et al., 2017a). Furthermore, BZP reduced the incidence of stroke, neuronal necrosis in the brain, cell swelling, and inflammatory infiltration in the kidneys of Dahl-ss hypertensive rats. These effects were related to antioxidant mechanisms. Based on the structural similarities between BZP and aspirin, we further explored the effects of BZP on platelet function. BZP selectively inhibited platelet aggregation induced by AA *in vitro*. However, BZP did not affect platelet aggregation induced by ADP and thrombin. Interestingly, BZP inhibited experimental thrombosis in rats *ex vivo*, suggesting that BZP could be used to treat and prevent stroke in Dahl-ss hypertensive rats through anti-platelet aggregation and anti-thrombus formation effects (Gao et al., 2017b). Because patients with cerebral ischemia require long-term treatment, and due to the convenience of oral preparations in clinics, enteric-coated pellets were synthesized by the College of Chemistry and Molecular Engineering of Zhengzhou University. Using an oral formulation, we further explored the preventive-effects and associated targets of BZP against ischemia-reperfusion injury in this study. Docking study results showed that BZP and C8E4 had similar structural abilities to inhibit 15-LOX-2 metabolism of AA. In this study, C8E4 was used as a positive control to further characterize the effects of BZP on focal cerebral ischemia-reperfusion injury and PC12 cell hypoxic injury induced by Na_2_S_2_O_4_. In this study, we observed neurobehavioral deficits, large infarct volumes, and severe brain swelling in MCAO rats. BZP improved nerve loss, reduced cerebral infarct volume, and reduced brain swelling in MCAO rats. In addition, we established an Na_2_S_2_O_4_-induced PC12 cell hypoxic injury model to observe changes in cell morphology and survival rate. The survival rate in the OGD/R injury group was significantly lower than that in the control group, and cell body size was reduced. BZP treatment improved the survival rate and morphology of hypoxic PC12 cells. These results indicated that BZP exerted protective effects against focal cerebral ischemia-reperfusion injury and PC12 cell hypoxic injury induced by Na_2_S_2_O_4_. Compared with C8E4 treatment, BZP significantly improved nerve loss, reduced cerebral infarct volume, and reduced brain swelling, indicating that BZP was more effective than C8E4. In addition, we further monitored cerebral blood flow dynamics, and confirmed that BZP increased cerebral blood flow, reduced infarct volume and swelling formation in a dose-dependent manner. (The data was not shown).

15-LOX is associated with a variety of diseases, including cerebrovascular diseases, hypertension, diabetes and obesity. The expression pattern of 15-LOX and the distribution of its metabolites are cell- and tissue-specific, and may play different roles in pathological processes (Singh and Rao, 2019). 15-LOX-2 acts on AA to produce 15-HETE, and 15-HETE generated under hypoxic conditions is mainly derived from 15-LOX-2. Previous studies showed that the expression of 15-LOX began to increase 12 h after cerebral ischemia, reached a peak at 48 h, and the gradually decreased at 72 h (Cui et al., 2010; Jin et al., 2013). During cerebral ischemia, 15-LOX may cause mitochondrial dysfunction, induce increased production of free radicals and lipid mediators, promote the release of cytokines and adhesion molecules, and induce apoptosis (van Leyen et al., 2006). Xiao et al. strongly indicate that BZP may serve as a potent and promising cardioprotective agent in treatment of ischemical reperfusion injury related injury, at least partially through targeting 12/15-LOX-2 (Xiao et al., 2019). Our experiments also showed that BZP could inhibit the production of 15-HETE *in vitro* and *in vivo*, similar to C8E4. Therefore, our study showed that BZP inhibited 15-LOX-2 and the results were the same as Xiao et al's research. 15-LOX is a key enzyme in the AA metabolic pathway and is involved in the inflammatory response (Ackermann et al., 2017). Our results suggested that BZP may act through inhibition of inflammation, therefore, we focused on the effects of BZP on inflammation.

The 15-LOX pathway induces inflammation through 15-HETE, and increased expression of IL-6, IFN-γ, IL-12, and TNF-α mRNA and protein (Miao et al., 2016). A previous study showed that the levels of TNF-α, IL-6, infarct volume, and cerebral edema increased in rats subjected to cerebral ischemia-reperfusion (Sears et al., 2009). 15-HETE could also promote production of inflammatory factors through activation of protein kinase C and mitogen-activated protein kinases. Inhibition of activity of these kinases resulted in decreased expression of IL-6 and TNF-α (Wen et al., 2007). Previous reports suggested that inhibition of 15-HETE production, or use of small interfering RNA of 15-LOX could reduce the infiltration of monocytes/macrophages and decrease the expression of intercellular adhesion molecules during hypoxia, thus reducing cell damage (Gutowska et al., 2012; Li et al., 2013). NF-κB is a nuclear transcription factor (Zhang et al., 2013), found in B lymphocyte progenitor cells, and is an intermediate hub of intracellular signal transduction. It participates in growth and development of the body and pathological processes. Recently, inflammatory cells and inflammatory mediators, and their roles in cerebral ischemia-reperfusion injury, have received extensive attention. The NF-κB transcriptional activation pathway is considered the main regulatory factor in inflammation and is an initiator of the inflammatory response and injury following cerebral ischemia (Harari and Liao, 2010; Minutoli et al., 2016). Cerebral ischemia results in activation of NF-κB in microglia which release inflammatory factors, such as TNF-α, ICAM-1, IL-6. Furthermore, iNOS and cyclooxygenase-2 (COX2) were activated after stroke, causing a strong inflammatory response (Wang et al., 2010; Fernandes et al., 2014; Lan et al., 2017). Reduction in the expression of NF-κB could reduce infarct volume, edema, and neurological dysfunction in rats with permanent cerebral ischemic injury (Huang et al., 2006). TNF-αis a potent pro-inflammatory chemokine. Chemokines attract neutrophils into ischemic tissues and stimulate the expression of IL-6 and intercellular adhesion molecules following ischemic stroke. Increased production of inflammatory factors, leads to upregulation of ICAM-1. In addition, activation of the TNF receptor activates the NF-κB signaling pathway, resulting in microglial to secretion of TNF-α, and further activation of microglia (Sriram et al., 2006). Inhibition of chemokine production could reduce cerebral edema and reduce cerebral infarction volume (Maria et al., 2012).We found that BZP significantly decreased the expression of NF-κB, TNF-α, IL-6, and ICAM-1 after ischemic stroke in rats and in PC12 cells subjected to hypoxic injury induced by Na_2_S_2_O_4_, suggesting that BZP might inhibit the production of 15-HETE, a metabolite of 15-LOX-2.

## Conclusion

Our results showed that BZP could improve focal cerebral ischemia-reperfusion injury in rats and PC12 cell injury induced by Na_2_S_2_O_4_ in dose/concentration-dependent manners. The mechanisms of these effects were associated with inhibition of production of 15-HETE and reduced expression of NF-κB, IL-6, TNF-α, and ICAM-1. Based on these results 15-LOX-2 might be a target of BZP against cerebral ischemia.

## Data Availability Statement

All datasets generated for this study are included in the article/supplementary material.

## Ethics Statement

The animal study was reviewed and approved by the Animal Ethics Committee of Zhengzhou University.

## Author Contributions

All authors (YG, XC, XZ, YW, HH, YM, JC) meet the essential authorship criteria required by the journal. YG, XC, XZ and YW performed the animal and cell experiment, analyzed data, interpreted results of experiments and prepared the manuscript; HH and YM provided the BZP compound for the whole experiment; JC designed the whole research and reviewed the final manuscript. All the authors have read and approved the final version.

## Conflict of Interest

The authors declare that the research was conducted in the absence of any commercial or financial relationships that could be construed as a potential conflict of interest.

## Abbreviations

BZP, Sodium (±)-5-bromo-2-(α-hydroxypentyl) benzoate, Brozopine; Br-NBP, 3-butyl-6-bromo-1(3H)-isobenzofuranone; NBP, 3-n-butylphthalide; C8E4, Tetraethylene glycol monooctyl ether; MCAO, The middle cerebral artery occlusion; Na^2^S^2^O^4^, Sodium hyposulfite; OGD, Oxygen and glucose deprivation; LOX, Lipoxygenase; 15-LOX-2, 15-Lipoxygenase-2; 15-HETE, 15-Hydroxyeicosatetraenoic acid; NF-κB, Nuclear factor-kappa B; TNF-α, Tumor Necrosis Factor; IL-6, Interleukin-6; ICAM-1Intercellular cell adhesion molecule-1, AA, Arachidonic acid; PBS, Phosphate-buffered saline; DMSO, Dimethyl sulfoxide.
